# Supplementary material for: A Systematic Review and Meta-analysis of the Prevalence and Impact of Pulmonary Bacterial Colonisation in Stable State Chronic Obstructive Pulmonary Disease (COPD)
Source: Biomedicines. 2021 Dec 31;10(1):81. doi: 10.3390/biomedicines10010081 (PMC8773377; doi:10.3390/biomedicines10010081)
Supplement: Supplementary file 1 [file biomedicines-10-00081-s001.zip › biomedicines-1523804-supplementary.pdf]

Supplementary File 1. Search strategies used for Ovid Medline, Embase, CINAHL, and Cochrane Central.

Ovid MEDLINE(R) ALL Search Strategy

1	copd.ti,ab.
2	chronic obstructive pulmonary disease.ti,ab.
3	chronic obstructive lung disease.ti,ab.
4	chronic obstructive airway? disease.ti,ab.
5	coad.ti,ab.
6	chronic respiratory disorder$.ti,ab.
7	chronic airflow obstruction.ti,ab.
8	smoking-related lung disease$.ti,ab.
9	exp Bronchitis, Chronic/
10	exp Pulmonary Disease, Chronic Obstructive/
11	chronic bronchitis.ti,ab.
12	emphysema.ti,ab.
13	1 or 2 or 3 or 4 or 5 or 6 or 7 or 8 or 9 or 10 or 11 or 12
14	carrier state.ti,ab.
15	exp Asymptomatic Infections/
16	asymptomatic infection$.ti,ab.
17	bacterial coloni*.ti,ab.
18	bacterial flora.ti,ab.
19	microflora.ti,ab.
20	exp Carrier State/
21	coloni*.ti,ab.
22	14 or 15 or 16 or 17 or 18 or 19 or 20 or 21
23	13 and 22
24	limit 23 to humans

Total results: 868

Embase Search Strategy

1	copd.ti,ab.
2	exp chronic obstructive lung disease/
3	chronic obstructive pulmonary disease.ti,ab.
4	Chronic obstructive lung disease.ti,ab.
5	Chronic obstructive airway$ disease.ti,ab.
6	coad.ti,ab.
7	chronic respiratory disorder$.ti,ab.
8	chronic airflow obstruction.ti,ab.
9	smoking-related lung disease$.ti,ab.
10	exp chronic bronchitis/
11	chronic bronchitis.ti,ab.
12	emphysema.ti,ab.
13	exp cigarette smoke-induced emphysema/ or exp emphysema/ or exp lung emphysema/
14	1 or 2 or 3 or 4 or 5 or 6 or 7 or 8 or 9 or 10 or 11 or 12 or 13
15	carrier state.ti,ab.
16	exp asymptomatic infection/
17	asymptomatic infection$.ti,ab.
18	exp bacterial colonization/
19	coloni*.ti,ab.
20	exp bacterial flora/
21	microflora.ti,ab.
22	flora.ti,ab.
23	exp microflora/
24	15 or 16 or 17 or 18 or 19 or 20 or 21 or 22 or 23
25	14 and 24
26	limit 25 to human

Total results: 3,251

CINAHL Search Strategy
Total results: 316

S25 S15 AND S24
S24 S16 OR S17 OR S18 OR S19 OR S20 OR S21 OR S22 OR S23
S23 microflora
S22 flora
S21 coloni*
S20 bacterial coloni#ation
S19 (MM "Bacterial Colonization")
S18 asymptomatic infection
S17 carrier state
S16 (MM "Carrier State")
S15 S1 OR S2 OR S3 OR S4 OR S5 OR S6 OR S7 OR S8 OR S9 OR S10 OR S11 OR S12 OR S13 OR S14
S14 emphysema
S13 (MM "Emphysema+")
S12 chronic bronchitis
S11 (MM "Bronchitis, Chronic")
S10 smoking#related lung disease
S9 chronic airflow obstruction
S8 chronic respiratory disorder*
S7 copd
S6 chronic obstructive airway* disease
S5 chronic obstructive lung disease
S4 chronic obstructive pulmonary disease
S3 copd
S2 (MM "Lung Diseases, Obstructive+")
S1 (MM "Pulmonary Disease, Chronic Obstructive+")

Cochrane CENTRAL Search Strategy

#1 MeSH descriptor: [Pulmonary Disease, Chronic Obstructive] explode all trees
#2 (chronic obstructive pulmonary disease):ti,ab
#3 (copd):ti,ab
#4 (chronic obstructive lung disease):ti,ab
#5 (chronic obstructive airway? disease):ti,ab
#6 (coad):ti,ab
#7 (chronic respiratory disorder):ti,ab
#8 (chronic airflow obstruction):ti,ab
#9 (smoking?related lung disease):ti,ab
#10 MeSH descriptor: [Bronchitis, Chronic] explode all trees
#11 (chronic bronchitis):ti,ab
#12 MeSH descriptor: [Emphysema] explode all trees
#13 (emphysema):ti,ab
#14 #1 or #2 or #3 or #4 or #5 or #6 or #7 or #8 or #9 or #10 or #11 or #12 or #13
#15 MeSH descriptor: [Carrier State] explode all trees
#16 (carrier state):ti,ab
#17 MeSH descriptor: [Asymptomatic Infections] explode all trees
#18 (asymptomatic infection):ti,ab
#19 (coloni?ation):ti,ab
#20 (coloni*):ti,ab
#21 MeSH descriptor: [Microbiota] explode all trees
#22 (flora):ti,ab
#23 (microflora):ti,ab
#24 #15 or #16 or #17 or #18 or #19 or #20 or #21 or #22 or #23
#25 #14 and #24

Total results: 133

Supplementary Figure S1

	D1	D2	D3	D4	D5	D6	D7	D8	D9	Overall
Andelid 2015	+	+	X	X	X	+	+		X	X
Banerjee 2004	-	+	X	+	+	+	+		+	+
Bogaert 2004	X	+	+	X	+	+	+		+	+
Cabello 1997a	+	+	X	+	+	+	+		+	+
Cabello 1997b	+	+	X	+	+	+	+		X	+
Einarsson 2016	-	+	X	+	+	+	+		+	+
Fruchter 2014	-	+	X	+	+	+	+		+	+
Garcia-Nunez 2014a	-	+	X	+	+	+	+		+	+
Garcia-Nunez 2014b	-	+	X	+	X	+	+		+	+
Hurst 2005	X	+	X	+	X	+	X		+	X
Jacobs 2018	+	+	+	X	+	+	+		+	+
Jordan 1976a	-	+	X	X	+	+	+		+	+
Jordan 1976b	-	+	X	X	+	+	+		+	+
Khurana 2014a	+	+	X	+	X	+	X		X	X
Khurana 2014b	+	+	X	+	X	+	X		X	X
Marin 2009a	-	+	X	+	+	+	+		X	+
Marin 2009b	-	+	X	+	+	+	+		X	+
Marin 2012	+	+	+	+	X	+	X		+	+
Mika 2018	+	+	X	+	+	+	+		X	+
Millares 2014	+	+	X	+	X	+	+		X	X
Miravitlles 2009a	+	+	+	+	X	+	+		+	+
Miravitlles 2009b	+	X	X	+	X	+	+		+	X
Miravitlles 2010	+	+	+	+	+	+	X		+	+
Monso 1995	-	+	X	+	+	+	+		+	+
Monso 1999	+	+	X	+	+	+	+		+	+
Patel 2002	-	+	X	+	+	+	+		+	+
Riise 1994a	-	+	X	+	X	+	+		X	X
Riise 1994b	-	+	X	+	X	+	+		X	X
Seemungal 2008	+	+	X	+	X	+	+		X	X
Sethi 2006	+	+	X	+	+	+	+		+	+
Sibila 2014	+	+	X	+	+	+	+		+	+
Sibila 2016	+	+	X	+	+	+	+		+	+
Simpson 2014	+	+	X	+	+	+	+		X	+
Simpson 2016	+	+	X	+	+	+	+		+	+
Singh 2014	+	+	+	+	+	+	+		+	+
Sriram 2018	+	+	X	+	+	+	+		+	+
Trudzinski 2018	+	+	X	X	+	+	+		+	+
Tumkaya 2006a	+	+	X	+	+	+	+		+	+
Tumkaya 2006b	+	+	X	+	+	+	+		+	+
Weinreich 2007	+	+	X	+	X	+	+		+	+
Wilkinson 2003a	+	+	X	+	X	+	+		+	+
Wilkinson 2003b	+	+	X	+	X	+	+		+	+
Wilkinson 2019a	+	+	+	+	+	+	+		+	+
Wilkinson 2019b	+	+	+	+	+	+	+		+	+
Zalacain 1999	+	+	+	+	+	+	+		+	+
Zhang 2010	+	+	X	+	+	+	+		+	+

D1: Was the sample frame appropriate to address the target population?
D2: Were study participants sampled in an appropriate way?
D3: Was the sample size adequate?
D4: Were the study subjects and the setting described in detail?
D5: Was the data analysis conducted with sufficient coverage of the identified sample?
D6: Were valid methods used for the identification of the condition?
D7: Was the condition measured in a standard, reliable way for all participants?
D8: Was there appropriate statistical analysis?
D9: Was the response rate adequate, and if not, was the low response rate managed appropriately?

Judgement
X High
- Unclear
+ Low
Not applicable

Figure S1. Risk of bias table summarising result from the Joanna Briggs Institute tool for assessing prevalence studies.
